# Vasodilator Responses of Perivascular Adipose Tissue-Derived Hydrogen Sulfide Stimulated with L-Cysteine in Pregnancy Hypertension-Induced Endothelial Dysfunction in Rats

**DOI:** 10.3390/antiox12111919

**Published:** 2023-10-26

**Authors:** Priscilla Bianca de Oliveira, Gabriela Palma Zochio, Edileia Souza Paula Caetano, Maria Luiza Santos da Silva, Carlos Alan Dias-Junior

**Affiliations:** 1 Department of Biophysics and Pharmacology, Institute of Biosciences, São Paulo State University (UNESP), Botucatu 18618-689, SP, Brazil; priscilla.bianca@unesp.br (P.B.d.O.); gabriela.zochio@unesp.br (G.P.Z.); edileia.paula@unesp.br (E.S.P.C.); mls.silva@unesp.br (M.L.S.d.S.); 2 Laboratory of Pharmacology, Marília Medical School (FAMEMA), Marília 17519-030, SP, Brazil

**Keywords:** hydrogen sulfide, perivascular adipose tissue, endothelial dysfunction, pregnancy hypertension

## Abstract

Endothelium-derived nitric oxide (NO)-induced vasodilation is impaired in pregnancy hypertension. However, the role of perivascular adipose tissue (PVAT)-derived hydrogen sulfide (H_2_S), as an alternative for counteracting vascular dysfunction, is incompletely clear in hypertensive disorders of pregnancy. Therefore, PVAT-derived H_2_S-induced vasodilation was investigated in pregnancy hypertension-induced endothelial dysfunction. Non-pregnant (Non-Preg) and pregnant (Preg) rats were submitted (or not) to the deoxycorticosterone (DOCA)-salt protocol and assigned as follows (*n* = 10/group): Non-Preg, Non-Preg+DOCA, Preg, and Preg+DOCA groups. Systolic blood pressure (SBP), angiogenesis-related factors, determinant levels of H_2_S (PbS), NO (NOx), and oxidative stress (MDA) were assessed. Vascular changes were recorded in thoracic aortas with PVAT and endothelium (intact and removed layers). Vasorelaxation responses to the substrate (L-cysteine) for the H_2_S-producing enzyme cystathionine-γ-lyase (CSE) were examined in the absence and presence of CSE-inhibitor DL-propargylglycine (PAG) in thoracic aorta rings pre-incubated with cofactor for CSE (pyridoxal-5 phosphate: PLP) and pre-contracted with phenylephrine. Hypertension was only found in the Preg+DOCA group. Preg+DOCA rats showed angiogenic imbalances and increased levels of MDA. PbS, but not NOx, showed increased levels in the Preg+DOCA group. Pre-incubation with PLP and L-cysteine elevated determinants of H_2_S in PVAT and placentas of Preg-DOCA rats, whereas no changes were found in the aortas without PVAT. Aortas of Preg-DOCA rats showed that PVAT-derived H_2_S-dependent vasodilation was greater compared to endothelium-derived H_2_S, whereas PAG blocked these responses. PVAT-derived H_2_S endogenously stimulated with the amino acid L-cysteine may be an alternative to induce vasorelaxation in endothelial dysfunction related to pregnancy hypertension.

## 1. Introduction

Strategies that aim to prevent or mitigate hypertensive disorders during pregnancy are being increasingly addressed by researchers; thus, a thin layer of fat that surrounds the important systemic arteries, named perivascular adipose tissue (PVAT), has become the target of all attention [1]. Studies suggest that PVAT may modulate vascular tone through the release of adipocyte-derived relaxing factors (ADRFs) under physiological conditions [2]. PVAT-derived factors have been shown to act as vasoactive agents that promote anticontractile effects [3]. Hence, reductions in vasoconstrictive responses induced by phenylephrine, angiotensin II, and serotonin have been found in systemic arteries with PVAT [4]. In normotensive pregnant rats, it has also been shown that PVAT plays an important role in controlling blood flow [5]. However, it remains unknown whether PVAT-released vasodilator factors are affected by endothelial dysfunction-associated pregnancy hypertension.

Endothelium-dependent vasodilation is also an essential mechanism for controlling blood pressure in healthy gestation [6]. Thereby, impaired endothelial function has been shown to be an aggravating factor for cardiovascular diseases, including gestational hypertension [7]. Previous evidence suggests that under endothelial dysfunction, the body activates compensatory mechanisms, in which PVAT may be of extreme relevance [8]. PVAT-dependent vasodilation may also involve the action of ADRFs through the activation of potassium channels; thus, it is essential to study molecules that act through this pathway and that are synthesized by PVAT, such as sulfide hydrogen (H_2_S) [9]. Importantly, H_2_S is produced by the endothelium, smooth muscle, and PVAT [9]. H_2_S was considered a gas with harmful effects, but recent studies indicate that H_2_S is an important molecule for stabilizing inflammatory processes and oxidative stress [10].

Studies demonstrate that PVAT-derived H_2_S plays a crucial role in cardiovascular homeostasis, while other findings have suggested that PVAT-derived H_2_S may also act negatively on cardiovascular diseases [11]. Interestingly, Golas et al. (2022) demonstrated that H_2_S synthesized by PVAT and the vascular wall had pro-contractile effects that may contribute to vascular dysfunction in hypertension in male rats [12]. However, the H_2_S from the vascular wall has also been shown to have a pro-relaxation effect, suggesting a compensatory mechanism for counteracting the dysfunctional vascular tone [12]. Accordingly, it has been suggested that the H_2_S produced by smooth muscle and the endothelium in pathological conditions may continue to exert a role in vascular homeostasis [13,14]. Regarding this, previous findings in male rats have demonstrated that H_2_S may be a PVAT-derived ADRF and that H_2_S may be involved in the anti-contractility presented on the face of stimulation with phenylephrine and serotonin [15,16]. The mechanism by which H_2_S exerts a vasodilator function seems to differ according to the anatomic location of the vessel [17,18].

Previous evidence suggests that H_2_S is synthesized enzymatically, involving three enzymes: cystathionine beta-synthase (CBS), cystathionine gamma-lyase (CSE), and 3-mercaptopyruvate sulfur transferase (3-MPST) [19]. Earlier studies have demonstrated that CSE-derived H_2_S synthesis may be stimulated by its substrate (L-cysteine) and cofactor (pyridoxal 5-phosphate) in the vascular system [20,21]. To demonstrate that H_2_S is synthesized by CSE in the PVAT, experimental studies have used an inhibitor of CSE, propargylglycine (PAG), and authors showed that PAG inhibited the anticontractile effects of PVAT, and reductions in the synthesis of H_2_S were also found in preadipocytes and adipocytes of rats, further confirming the extreme importance of PVAT-derived H_2_S in vasodilation [15,22,23]. However, vasodilatory effects dependent on PVAT induced by H_2_S stimulation in pregnancy hypertension-associated endothelial dysfunction warrant further investigation.

Recent findings suggest that, in physiological situations, H_2_S is elementary in the maintenance of vascular tone and that H_2_S may even be conceptualized as an essential ADRF [24]. Moreover, in healthy pregnancy, there are marked transformations in the arrangement of adipose tissue [25] and several other physiological adaptations, including reductions in systemic vascular resistance [26]. Hence, it is extremely relevant to understand how the PVAT-derived H_2_S regulates vascular tonus in gestational hypertension, which is the focus of the present study.

We and others have examined the role of PVAT-derived H_2_S in anticontractility, and in healthy pregnancy, there were no disturbances in PVAT [1,27]. Moreover, in pregnancy hypertension-induced endothelial dysfunction, the PVAT had its anticontractile effect enhanced by H_2_S stimulation, while the endothelium showed no anticontractile response, and the anticontractile effect attributed to PVAT was dependent on ATP-sensitive potassium channels [27]. However, the PVAT-derived H_2_S-dependent vasodilation is still unclear in endothelial dysfunction induced by pregnancy hypertension.

Therefore, in search of understanding the vascular function focusing on vasorelaxation in the face of stimulation in the synthesis of PVAT-derived H_2_S, the objective of this study was to evaluate the vasodilator responses of the PVAT-derived H_2_S under endothelial dysfunction induced by gestational hypertension.

## 2. Materials and Methods

### 2.1. Experimental Design

The experimental protocol was approved by the Ethics Committee on the Use of Animals (CEUA), Institute of Biosciences of Botucatu (IBB), São Paulo State University (CEUA/IBB, protocol number: 1083/2018, June, 2018), which complied with the Animal Research: Reporting of In Vivo Experiments (ARRIVE) guidelines and with the U.K. Animals (Scientific Procedures) Act, 1986 and associated guidelines, and EU Directive 2010/63/EU for animal experiments. All experiments were carried out and analyzed by blind and trained operators.

Age-timed male and female Wistar rats (200–250 g) from our institutional *vivarium* were used. Animals were housed in cages on a 12 h light/dark cycle (at 22 ± 2 °C) and had free access to rat chow and drinking water.

Female rats (*n* = 10 per group) were equally randomized and assigned as follows: non-pregnant rats not submitted to the DOCA-salt protocol (Non-Preg group), non-pregnant rats submitted to the DOCA-salt protocol (Non-Preg+DOCA), pregnant rats not submitted to the deoxycorticosterone acetate (DOCA)-salt protocol (Preg group), and pregnant rats submitted to the DOCA-salt protocol (Preg+DOCA group).

Female virgin rats were used in the Non-Preg and Non-Preg+DOCA groups. In the Preg and Preg+DOCA groups, each female virgin rat was separately mated overnight. The 1st day of pregnancy was defined as the day on which the spermatozoa were found in a vaginal smear containing anucleated cornified squamous cells.

The DOCA-salt protocol consisted of three intraperitoneal (i.p.) injections of DOCA (#D7000; Sigma, St. Louis, MO, USA) and the replacement of drinking water by saline (0.9% NaCl) solution throughout rat pregnancy.

Preg+DOCA rats received an i.p. injection of DOCA (12.5 mg/kg) in depot form on the first day of pregnancy and i.p. injections of DOCA (6.25 mg/kg) on the seventh and fourteenth days of pregnancy. Preg rats received i.p. injections of saline on the respective days corresponding to the Preg+DOCA group.

Non-Preg+DOCA rats received an i.p. injection of the first dose of DOCA (12.5 mg/kg) and i.p. injections of DOCA (6.25 mg/kg) on the seventh and fourteenth days later. Non-Preg rats received i.p. injections of saline on the respective days corresponding to the Non-Preg+DOCA group.

The DOCA-salt protocol induces hypertension in pregnancy in rodents, as previously described [28,29].

### 2.2. Euthanasia, Blood Sampling, and Fetal and Placental Parameters

Preg and Preg+DOCA rats were weighed and euthanized on the nineteenth day of pregnancy. Non-Preg and Non-Preg+DOCA rats were weighed and euthanized on the nineteenth day after starting the protocol. All animals were euthanized with an isoflurane overdose (5%) followed by exsanguination.

After euthanasia, all rats underwent a cesarean section to access the uterus. Then, the individual weights of fetuses and placentas, and the total number of pups were recorded.

Blood samples were collected in lyophilized heparin-containing tubes (Vacutainer; Becton Dickinson, Oxford, UK) and immediately centrifuged for plasma separation.

Plasma, placenta, and thoracic aorta segments were stored at −80 °C until used for biochemical analysis. The thoracic aorta segment was excised and placed in cold Krebs solutions to perform the vascular reactivity experiments.

### 2.3. Systolic Blood Pressure Measurements

Systolic blood pressure was measured using tail-cuff plethysmography (Insight, Ribeirão Preto, São Paulo, Brazil, catalog #EFF 306) on the 1st (baseline), 14th, and 19th days of pregnancy in the Preg and Preg+DOCA groups, and on the 1st day of the protocol (baseline) and the 14th and 19th days after starting the protocol in the Non-Preg and Non-Preg+DOCA groups.

To record systolic blood pressure measurements, conscious rats were first acclimated, for 10 min, in a quiet room, conditioned, and contained in heated boxes (Insight, Ribeirão Preto, São Paulo, Brazil, catalog #EFF 307). Systolic blood pressure was determined as the mean of cuff inflation–disinflation cycles (three measurements).

### 2.4. Vascular Reactivity Experiments

The thoracic aorta was dissected and cut into 8 rings of 4 mm in length, totaling 80 aortic rings from each group. Then, we analyzed the vascular function as follows: an aortic ring with intact PVAT and endothelium (+PVAT+E), an aortic ring with intact PVAT and denuded endothelium (+PVAT-E), an aortic ring without PVAT and intact endothelium (-PVAT+E), and an aortic ring without PVAT and endothelium (-PVAT-E).

With the aid of a dissection microscope, the surrounding PVAT was carefully dissected and separated from the thoracic aorta. The endothelium was carefully removed by scraping (5 times) the interior of the thoracic aorta around the tip of the forceps.

Thoracic aortic rings were placed in a 10 mL organ bath chamber containing Krebs-Henseleit nutrient solution (in mmol/L): NaCl 130; KCl 4.7; CaCl_2_ 1.6; KH_2_PO_4_ 1.2; MgSO_4_ 1.2; NaHCO_3_; glucose 11.1; gassed continuously with a mixture of 95% O_2_/5% CO_2_; and maintained at 37 °C (pH 7.4). The thoracic aorta rings were suspended between two wire hooks. One hook was attached to a fixed support and the other hook was connected to an isometric force transducer. All thoracic aorta rings were stretched until an optimal basal tension of 1.5 g, which was determined by length–tension relationship experiments [30,31]. Then, aortic rings were allowed to equilibrate for 60 min, with the bath physiological solution being changed every 15–20 min. Changes in the tension of thoracic aorta rings were recorded using FORT10 isometric force transducers connected to a PC-based MP100 System and analyzed offline, using AcqKnowledge software (version 3.5.7, Biopac Systems Inc., Goleta, CA, USA). After the equilibration, each thoracic aorta ring was challenged to potassium chloride (KCl; 96 mM) as a test contraction and viability of the tissue. Once a reproducible and maximum KCl contraction was reached, all aortic rings were washed out (3 times) with Krebs–Henseleit solution (10 min intervals each), and the aortic rings returned to the baseline tension. Then, endothelium removal was determined by the absence of acetylcholine (ACh)-induced relaxation in the preparations +PVAT-E and -PVAT-E. PVAT removal was confirmed by the lower concentrations of phenylephrine (Phe) required to achieve the level of contractile tone observed in the thoracic aorta rings with PVAT, which was consistent with the anticontractile effect of PVAT, as previously demonstrated [27]. Each aortic ring was rinsed with Krebs–Henseleit physiological solution 3–5 times (10 min each) to avoid biases possibly caused by the KCl and ACh used.

To assess the vasorelaxation induced by PVAT and the endothelium, thoracic aorta rings were pre-contracted with phenylephrine (Phe; 10^−6^ M). Once a steady state was reached, a pre-incubation (10 min) with pyridoxal-5 phosphate (PLP; 2 mM) was performed. Then, a concentration–response curve was performed using L-cysteine (L-Cys; 10^−9^–10^−4^ M).

To investigate the involvement of CSE-synthesized H_2_S, thoracic aorta rings were pre-contracted with Phe (10^−6^ M). Once a steady state was reached, a pre-incubation (10 min) with PLP (2 mM) in the presence of DL-propargylglycine (PAG; 1 mM) was made. Then, a cumulative concentration curve of L-Cys (10^−9^–10^−4^ M) was assessed.

To examine the involvement of the endothelium in vasorelaxant responses, the thoracic aorta rings were pre-contracted with Phe (10^−6^ M). Once a plateau state was noticed, a cumulative concentration curve using ACh (10^−9^–10^−4^ M) was performed.

To assess the contribution of NO synthesized by the NO synthase, thoracic aorta rings were pre-incubated (10 min) with the inhibitor of the NO synthase Nω-Nitro-L-arginine methyl ester (L-NAME) hydrochloride (10^−4^ M). Then, thoracic aorta rings were pre-contracted with Phe (10^−6^ M). Once a steady state was reached, a cumulative concentration curve using ACh (10^−9^–10^−4^ M) was assessed.

Anticontractile effects of PVAT and the endothelium were also examined. In this experimental setup, a pre-incubation (10 min) with PLP (2 mM) and L-Cys (10 mM) was performed. Then, a cumulative concentration curve using Phe (10^−12^–10^−4^ M) was assessed.

### 2.5. Determination of Fms-like Tyrosine Kinase 1 (sFlt-1) and Placental Growth Factor (PLGF) Levels in Plasma

Plasma levels of anti-angiogenic factor sFlt-1 (#ELM-VEGFR1, RayBiotech Inc., Norcross, GA, USA) and angiogenic factor PLGF (#E-EL-R0742, Elabscience Inc., Houston, TX, USA) were determined according to the manufacturer’s instructions of the commercial enzyme-linked immunoassay (ELISA) kits. Plasma levels of sFlt-1 and PLGF were expressed in pg/mL.

### 2.6. Determination of H_2_S in Plasma

Determinants of H_2_S levels in plasma were analyzed, as previously described [32,33]. Briefly, plasma samples (75 μL) were mixed with zinc acetate 1% (250 μL) and distilled water (425 μL). In this mixture, 150 μL (20 mmol/L) of N-dimethyl-p-phenylenediamine sulfate in HCl (7.2 M) and 150 μL (30 mmol/L) of FeCl_3_ in HCl (1.2 M) were added. Then, an incubation (10 min), at room temperature, with trichloroacetic acid 10% (250 μL) was added to remove proteins, and the reaction mixture was centrifuged (15 min) at 12,000× *g*. The absorbance of the resulting supernatant (200 μL) was read (670 nm) in a spectrophotometer (Synergy 4, BioTek, Winooski, VT, USA) in a 96-well microplate. The concentration of H_2_S in the solution was calculated against a calibration curve (3.13–100 μM) of sodium hydrosulfide (NaHS). Determinants of H_2_S levels were expressed as µmol/L.

### 2.7. Determination of NO Metabolites in Plasma

NO metabolites were examined with Griess reagents, as previously described [34]. Briefly, plasma (190 µL) was deproteinized by adding zinc acetate (10 µL) solution (300 g/L) and centrifuged (30 min) at 10,000× *g* (4 °C). In a microplate, 50 μL of plasma supernatant was incubated (3 h) at 37 °C with 50 μL of sulfanilamide (2%, *m*/*v*), 50 μL (0.1%, *v*/*v*) of N-(1-naphthyl)-ethylenediamine dihydrochloride—NED), and 100 μL of vanadium chloride III (5%, *m*/*v*) solution in a mixer (Eppendorf, Darmstadt, Germany) under agitation and in the absence of light. A calibration curve (1.56–100 µmol/L) was pipetted by incubating prepared nitrite solutions with deionized water and Griess reagents. Absorbance was read at 535 nm in a spectrophotometer (Synergy 4, Biotek, Winooski, VT, USA). Nitrite and nitrate (NOx) levels were expressed as µmol/L.

### 2.8. Determinants of Oxidative Stress in Plasma

Lipid peroxidation was determined using the measurements of thiobarbituric acid (TBA)–reactive species (TBARS), as previously described [35]. Briefly, TBA reacts with the end product of lipid peroxidation malondialdehyde (MDA), and a colorimetric reaction is produced. This product is read (at 532 nm) in a spectrophotometer (Synergy 4, Biotek, Winooski, VT, USA). Briefly, plasma samples (100 μL) were added in testing tubes containing a reaction mixture of distilled water (100 μL), 50 μL of sodium dodecyl sulfate (SDS, 8.1%), 375 μL of acetic acid (20%), and 375 μL of TBA (0.8%), and incubation was performed in a water bath (1 h) at 95 °C. Then, testing tubes were centrifuged (10 min) at 15,000× *g*. The standard curve was prepared in a similar manner, in which plasma samples were replaced by 25 μL of MDA (known concentrations). Plasma levels of TBARS were calculated against a standard curve of MDA (20–320 nmol). The results were expressed in mmol/mL.

### 2.9. Determination of H_2_S Levels in PVAT, The Aorta without PVAT, and the Placenta

Determinants of H_2_S levels in tissue samples were assessed using the method of lead sulfide (PbS) formation, as previously described [36,37]. The PVAT, the aorta without PVAT, and the placentas were excised, homogenized in cold sterile phosphate buffer saline (PBS), and centrifuged (10 min) at 10,000× *g* (4 °C). In a microplate, supernatants (0.2 mg/mL protein) diluted with PBS (100 mM, pH 7.4) were mixed with CSE cofactor PLP (2 mM) and substrate L-Cys (10 mM). A filter paper was used to cover the microplate, which was pre-embedded with lead (Pb) acetate (100 mM). Then, the covered microplate was allowed to dry (3 h) at 37 °C. The PbS formed a dark brown precipitate, and dark spots on the filter paper were read by the optical density, analyzed, and quantified using the ImageJ software (NIH, Bethesda, MD, USA). To calculate the concentrations, a calibration curve (7.8–500 mM) was generated with the H_2_S donor (NaHS). Determinants of H_2_S levels in tissue samples were expressed as nmol/mg protein.

### 2.10. Statistical Analysis

Shapiro–Wilk tests were applied to verify the normality of data distribution, using the GraphPad Prism^®^ (8.0) software (San Diego, CA, USA). Student’s *t*-tests were used, and one-way analysis of variance (ANOVA) and repeated-measure two-way ANOVA followed by Tukey’s post hoc tests were applied for multiple comparisons. A value of probability (*p*) < 0.05 was considered statistically significant. The results are expressed as mean ± SEM.

### 2.11. Drugs and Chemicals

Deoxycorticosterone acetate (DOCA, D7000), L-cysteine (w326305), pyridoxal 5-phosphate (P9255), DL-propargylglycine (P7888), acetylcholine (A6625), L-NAME (N5751), phenylephrine (P6126), and sodium hydrosulfide (NaHS-161527) were used in this study.

DOCA was dissolved in propylene glycol (1883), whereas all other compounds were dissolved in distilled water. Propylene glycol was purchased from Dinamica Chemicals (São Paulo, Brazil), and other compounds were purchased from Sigma Aldrich (St. Louis, MO, USA).

## 3. Results

### 3.1. Systolic Blood Pressure Measurements in Non-Pregnant and Pregnant Rats Submitted (or Not) to the DOCA-Salt Protocol

**Figure 1:**
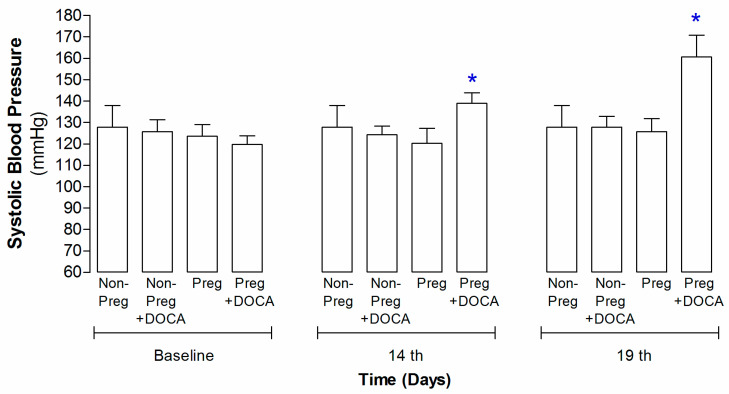
Systolic blood pressure in Non-Preg, Non-Preg+DOCA, Preg, and Preg+DOCA groups. Values are means ± SEM. Symbols indicate significant differences (* *p* < 0.05) in the Preg+DOCA group versus the respective baseline.

Baseline values showed no differences in the systolic blood pressure measurements, while significant increases were recorded on pregnancy days 14 and 19 of the Preg+DOCA group compared to the other groups (Figure 1; Supplementary Table S1).

### 3.2. Placental and Fetal Parameters of Pregnant Rats Submitted (or Not) to the DOCA-Salt Protocol

**Figure 2:**
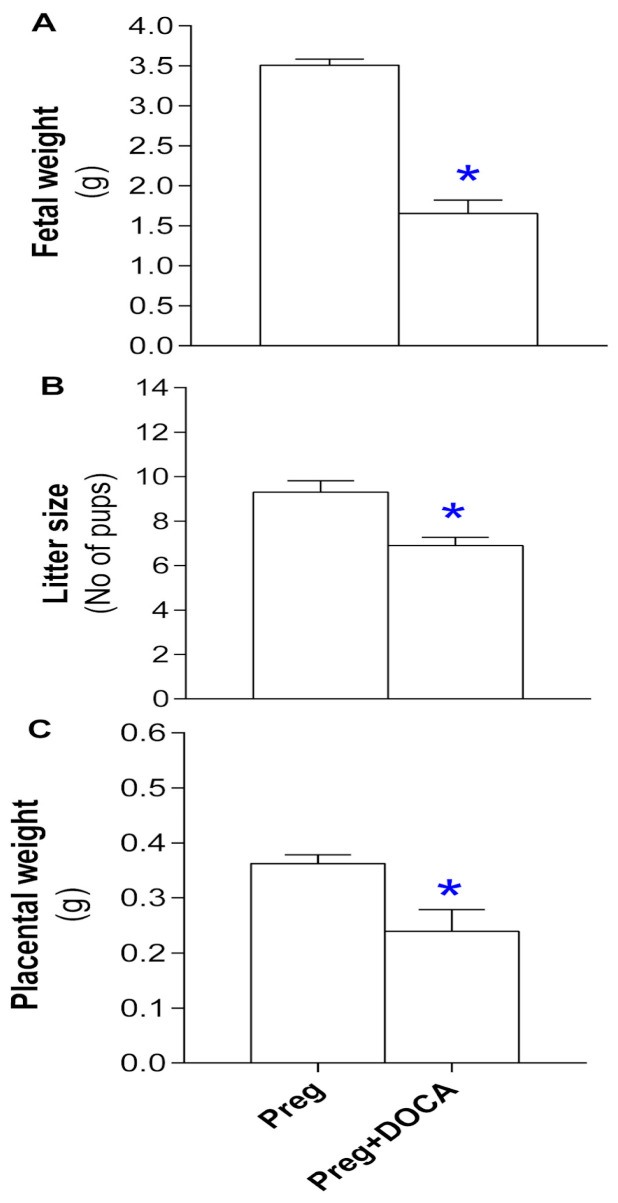
Fetal weight (**A**), total number of pups (litter size, (**B**)), and placental weight (**C**) of the Preg and Preg+DOCA groups. Values are means ± SEM. Symbols indicate significant differences (* *p* < 0.05) versus Preg group.

Pregnant rats submitted to the DOCA-salt protocol clearly showed significant reductions in fetal and placental weights and decreased number of pups in the Preg+DOCA group compared to the Preg group (Figure 2; Supplementary Table S2).

### 3.3. Angiogenesis Biomarker Levels in Plasma from Non-Pregnant and Pregnant Rats Submitted (or Not) to the DOCA-Salt Protocol

**Figure 3:**
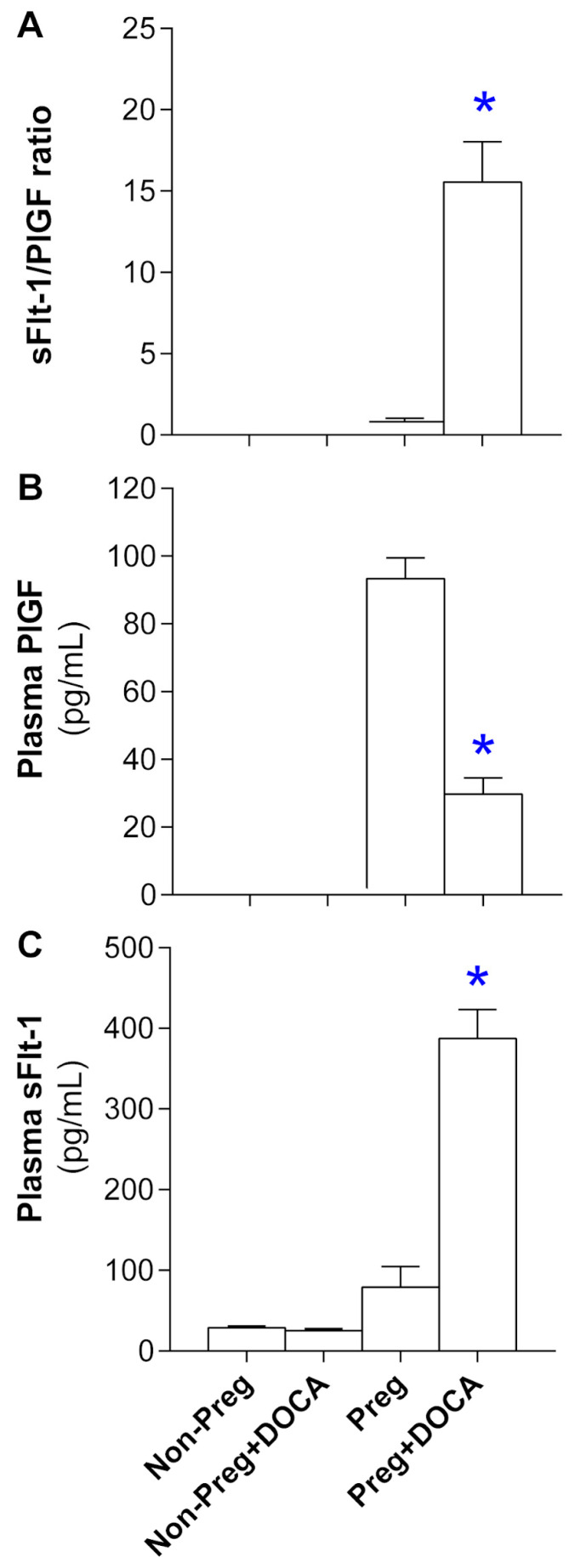
Levels of anti-angiogenic (soluble fms-like tyrosine kinase-1: sFlt-1) and angiogenic (placental growth factor: PlGF) factors in plasma. sFlt-1/PlGF ratio (**A**), plasma levels of PlGF (**B**), and sFlt-1 (**C**) in Non-Preg, Non-Preg+DOCA, Preg, and Preg+DOCA groups. Values are means ± SEM. Symbols indicate significant differences (* *p* < 0.05) versus Preg group.

The sFlt-1/PLGF ratio was calculated and was greater in the Preg+DOCA rats compared to the Preg group (Figure 3A; Supplementary Table S2). Plasma PLGF levels were lower in the Preg+DOCA rats compared to the Preg group, while both non-pregnant groups showed no PLGF in plasma (Figure 3B; Supplementary Table S2). Plasma sFlt-1 levels were higher in the Preg+DOCA group compared to the Preg group (Figure 3C; Supplementary Table S2).

### 3.4. Plasma Level Determinants of H_2_S, NO, and Oxidative Stress in Non-Pregnant and Pregnant Rats Submitted (or Not) to the DOCA-Salt Protocol

**Figure 4:**
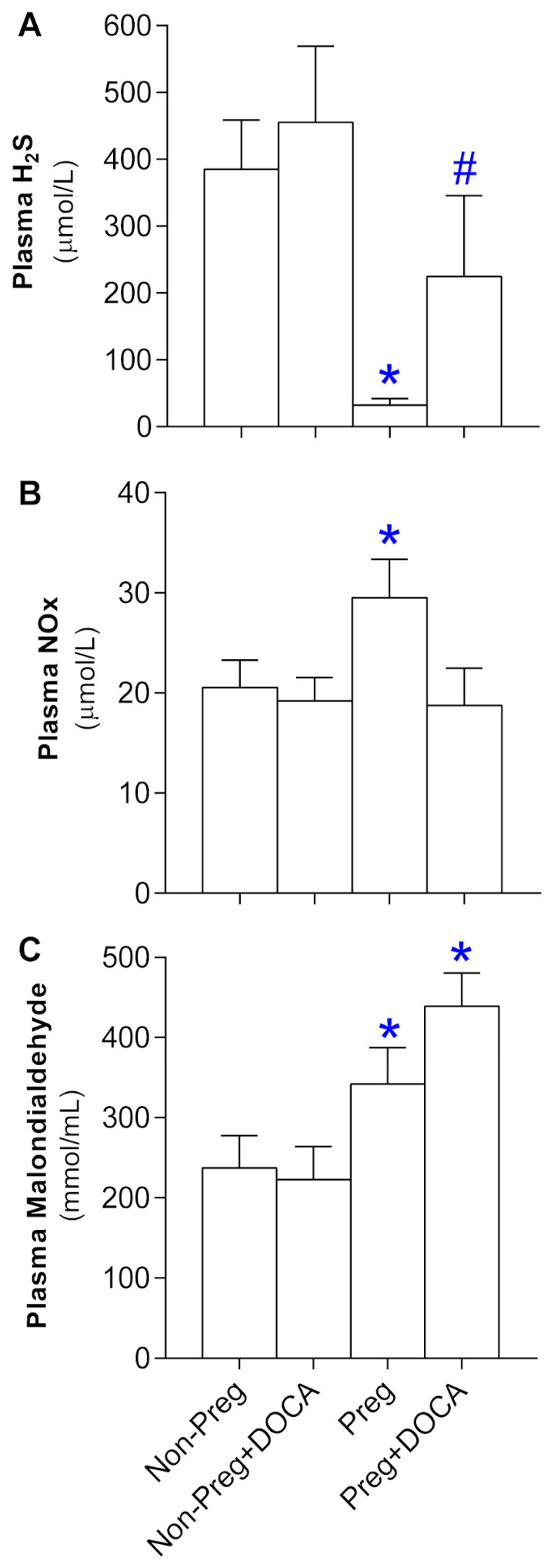
Plasma level determinants of H_2_S (**A**), NO (**B**), and oxidative stress (**C**) in Non-Preg, Non-Preg+DOCA, Preg, and Preg+DOCA groups. Values are means ± SEM. Symbols indicate significant differences (* *p* < 0.05) *versus* Non-Preg and Non-Preg+DOCA groups (**A**); # *p* < 0.05 versus Preg group (**A**); * *p* < 0.05 *versus* other groups (**B**), and * *p* < 0.05 versus Non-Preg and Non-Preg+DOCA groups (**C**).

Reductions in plasma level determinants of H_2_S were found in the Preg group compared to the non-pregnant groups, and elevated levels were found in the Preg+DOCA group compared to the Preg group (Figure 4A; Supplementary Table S2). Plasma levels of NO metabolites showed a significant increase in the Preg group compared to the others (Figure 4B; Supplementary Table S2). Plasma levels of MDA were higher in both Preg and Preg+DOCA groups compared to the others (Figure 4C; Supplementary Table S2).

### 3.5. Stimulated H_2_S Synthesis in the PVAT, the Aorta without PVAT, and the Placenta

**Figure 5:**
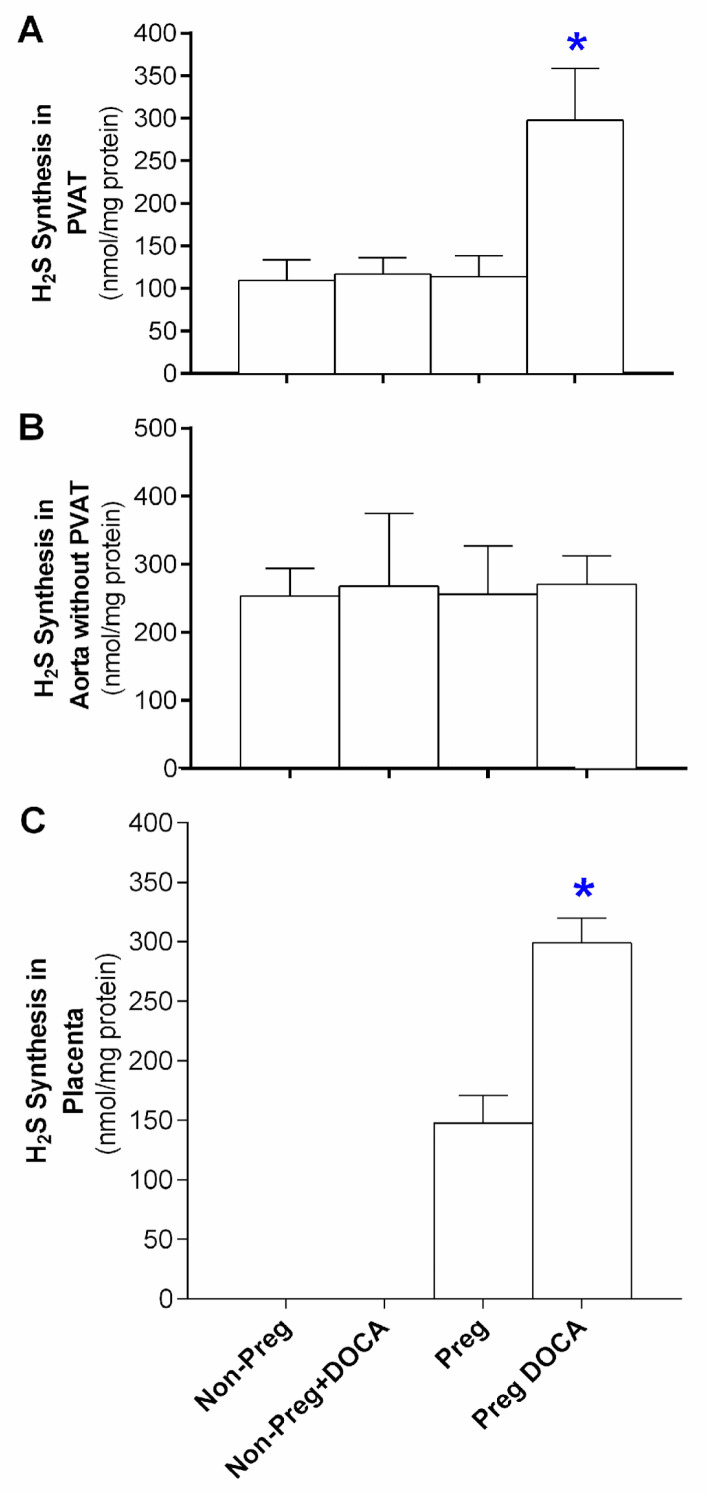
Level determinants of H_2_S in PVAT (**A**) and aorta without PVAT (**B**) from Non-Preg, Non-Preg+DOCA, Preg, and Preg+DOCA groups. Level determinants of H_2_S in the placenta of the Preg and Preg+DOCA groups. Values are means ± SEM. Symbols indicate significant differences (* *p* < 0.05) *versus* other groups (**A**) and * *p* < 0.05 versus Preg group (**C**).

Incubation with PLP and L-cysteine showed increases in the PVAT level determinants of H_2_S in the Preg+DOCA group compared to the others (Figure 5A), while no significant differences among all groups were found in the aortas without PVAT (Figure 5B). Increases in placental level determinants of H_2_S were found in the Preg+DOCA group compared to the Preg group (Figure 5C).

### 3.6. Vasodilation Responses via Endogenous H_2_S Stimulation in Aortas with Intact (or Removed) PVAT and/or Endothelium from Non-Pregnant and Pregnant Rats Submitted (or Not) to the DOCA-Salt Protocol

**Figure 6:**
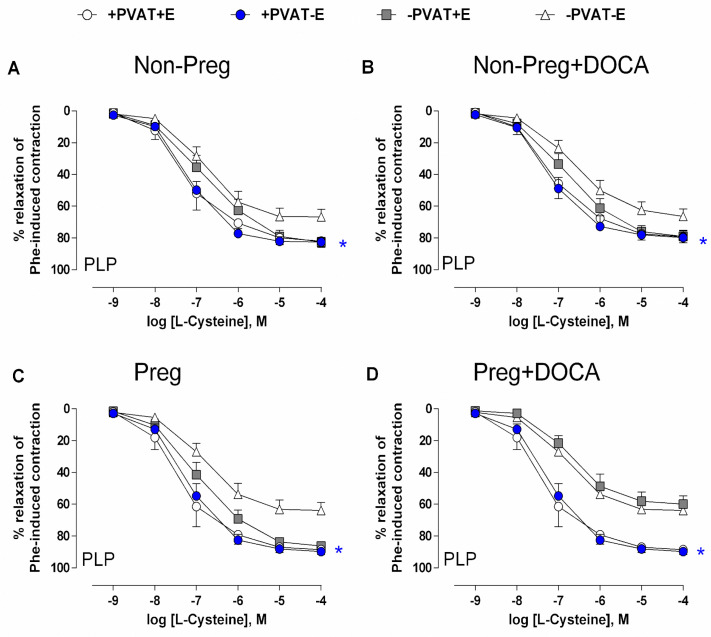
Concentration-dependent vasodilation responses to L-cysteine, after pre-incubation with PLP and pre-contracted with phenylephrine, in thoracic aortic rings with intact PVAT and endothelium (+PVAT+E), intact PVAT and endothelium removed (+PVAT−E), PVAT removed and intact endothelium (−PVAT+E), and both PVAT and endothelium removed (−PVAT−E) from Non-Preg group (**A**), Non-Preg+DOCA group (**B**), Preg group (**C**), and Preg+DOCA group (**D**). Values are means ± SEM. Symbols indicate significant differences (* *p* < 0.05) *versus* the respective aortic rings −PVAT−E (**A**–**C**); significant differences (* *p* < 0.05) *versus* the respective aortic rings −PVAT+E and −PVAT−E (Figure 6D).

L-cysteine, the substrate for H_2_S synthesis, showed similar concentration-dependent vasodilation responses in aortic rings with PVAT and endothelium, without endothelium, and without PVAT, whereas lower and similar vasorelaxation responses were found in the aortic rings without PVAT and endothelium from the Non-Preg, Non-Preg+DOCA, and Preg groups (Figure 6A–C; Supplementary Table S3).

In aortic rings from the Preg+DOCA group in which PVAT was preserved, L-cysteine-induced vasorelaxation maximal responses were significantly higher compared to the aortas without PVAT in which the endothelium was preserved or removed (Figure 6D; Supplementary Table S3).

### 3.7. Vascular Responses to L-Cysteine in the Presence of H_2_S Synthesis Inhibitor in Aortas with Intact (or Removed) PVAT and/or Endothelium from Non-Pregnant and Pregnant Rats Submitted (or Not) to the DOCA-Salt Protocol

**Figure 7:**
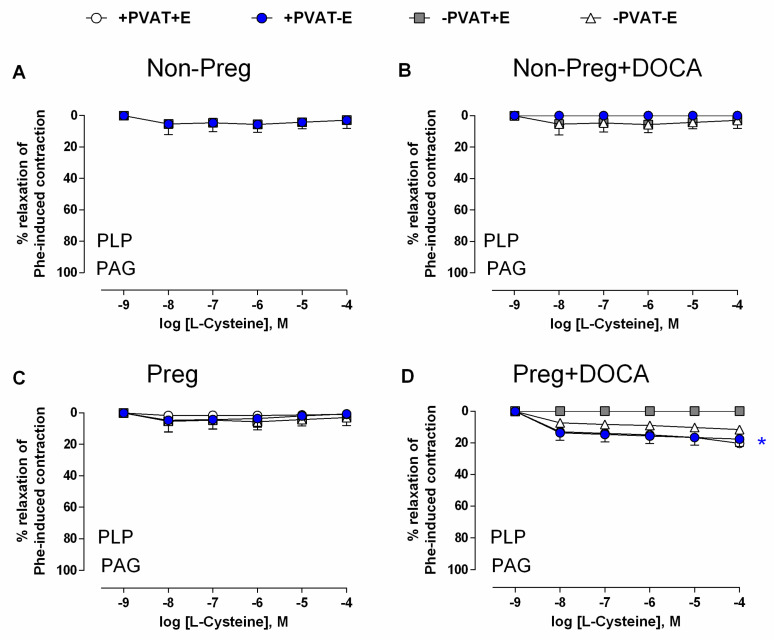
Concentration-dependent responses to L-cysteine, after pre-incubation with PLP and CSE inhibitor (PAG), and pre-contracted with phenylephrine, in thoracic aortic rings with intact PVAT and endothelium (+PVAT+E), intact PVAT and endothelium removed (+PVAT−E), PVAT removed and intact endothelium (−PVAT+E), and both PVAT and endothelium removed (−PVAT−E) from Non-Preg group (**A**), Non-Preg+DOCA group (**B**), Preg group (**C**), and Preg+DOCA group (**D**). Values are means ± SEM. Symbol (*) indicates significant differences (* *p* < 0.05) for aortic rings +PVAT+E and +PVAT−E *versus* the respective aortic ring −PVAT+E (Figure 7D).

Vasorelaxation responses to L-cysteine in all aortic rings from the Non-Preg, Non-Preg+DOCA, and Preg groups were inhibited in the presence of the CSE inhibitor PAG (Figure 7A–C; Supplementary Table S3). 

Aortic rings without PVAT and intact endothelium from the Preg-DOCA group showed total blockage of L-cysteine-induced vasorelaxation in the presence of PAG, while the other three preparations of aortic rings with PVAT and endothelium, with PVAT and endothelium removed, and without PVAT and endothelium elicited significant and weaker L-cysteine-induced vasodilation (Figure 7D; Supplementary Table S3).

### 3.8. Vasodilation Responses via Endogenous NO Stimulation in Aortas with Intact (or Removed) PVAT and/or Endothelium from Non-Pregnant and Pregnant Rats Submitted (or Not) to the DOCA-Salt Protocol

**Figure 8:**
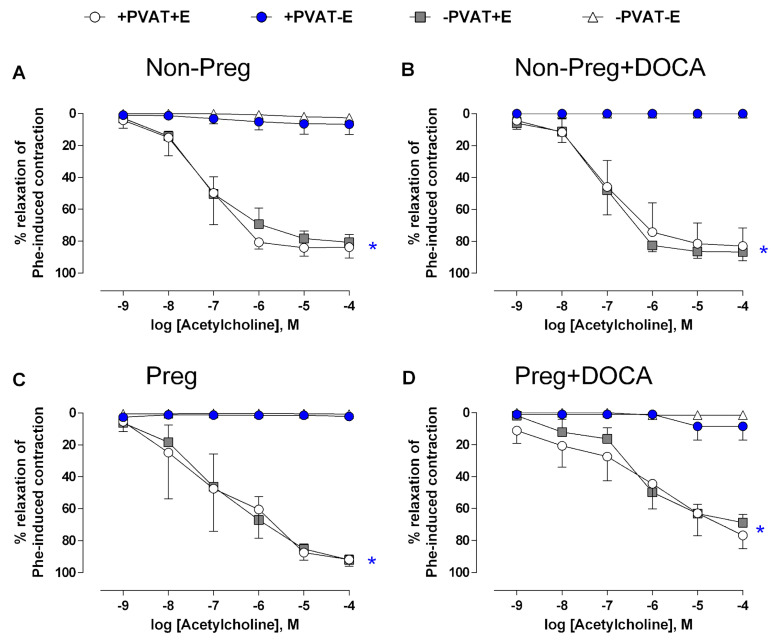
Concentration-dependent vasodilation responses to acetylcholine, after pre-contracted with phenylephrine, in thoracic aortic rings with intact PVAT and endothelium (+PVAT+E), intact PVAT and endothelium removed (+PVAT−E), PVAT removed and intact endothelium (−PVAT+E), and both PVAT and endothelium removed (−PVAT−E) from Non-Preg group (**A**), Non-Preg+DOCA group (**B**), Preg group (**C**), and Preg+DOCA group (**D**). Values are means ± SEM. Symbols indicate significant differences (* *p* < 0.05) *versus* the respective aortic rings +PVAT−E and −PVAT−E (**A**–**D**).

Acetylcholine was used to activate NO synthase and induce NO-dependent vasorelaxation. Aortic rings with intact endothelium in which PVAT was preserved or removed from Non-Preg, Non-Preg+DOCA, and Preg groups showed similar maximal vasodilation responses (Figure 8A–C; Supplementary Table S3). However, aortic rings with intact PVAT and denuded endothelium showed a total absence of response, which was similar to that observed in aortic rings without PVAT and endothelium (Figure 8A–C; Supplementary Table S3).

Aortic rings from the Preg+DOCA group showed that endothelium-dependent maximal responses were significantly impaired in intact endothelium aortas with PVAT and without PVAT, while aortic rings in which the endothelium was removed and PVAT was preserved showed no significant response, which was similar to what was observed in aortic rings without PVAT and endothelium (Figure 8D; Supplementary Table S3).

### 3.9. Vascular Responses to Acetylcholine in The Presence of NO Synthesis Inhibitor in Aortas with Intact (or Removed) PVAT and/or Endothelium from Non-Pregnant and Pregnant Rats Submitted (or Not) to the DOCA-Salt Protocol

**Figure 9:**
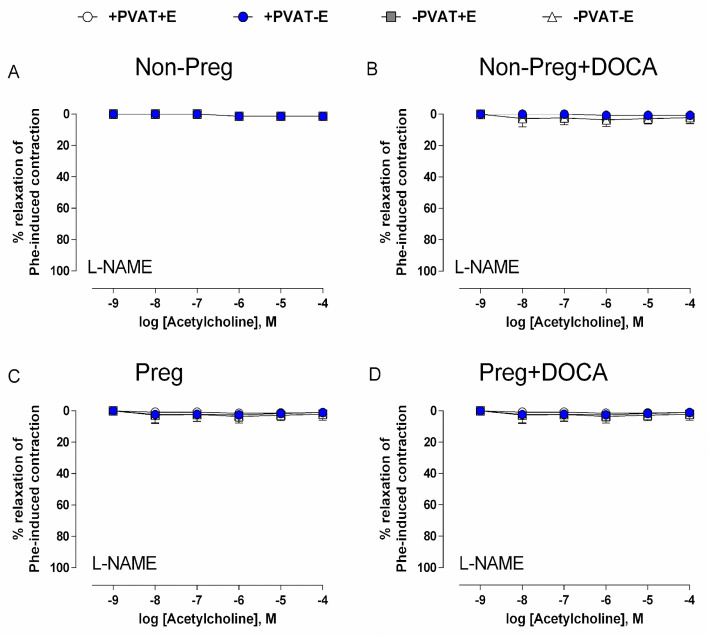
Concentration-dependent vasodilation responses to acetylcholine, after pre-incubation with NO synthase inhibitor (L-NAME) and pre-contracted with phenylephrine, in thoracic aortic rings with intact PVAT and endothelium (+PVAT+E), intact PVAT and endothelium removed (+PVAT−E), PVAT removed and intact endothelium (−PVAT+E), and both PVAT and endothelium removed (−PVAT−E) from Non-Preg group (**A**), Non-Preg+DOCA group (**B**), Preg group (**C**), and Preg+DOCA group (**D**). Values are means ± SEM.

In the presence of the NO synthase inhibitor L-NAME, acetylcholine-induced responses were not found in aortic rings from the Non-Preg, Non-Preg+DOCA, Preg, and Preg+DOCA groups (Figure 9A–D).

### 3.10. Anticontractile Responses Induced by PVAT and Endothelium in Aortas from Non-Pregnant and Pregnant Rats Submitted (or Not) to the DOCA-Salt Protocol

**Figure 10:**
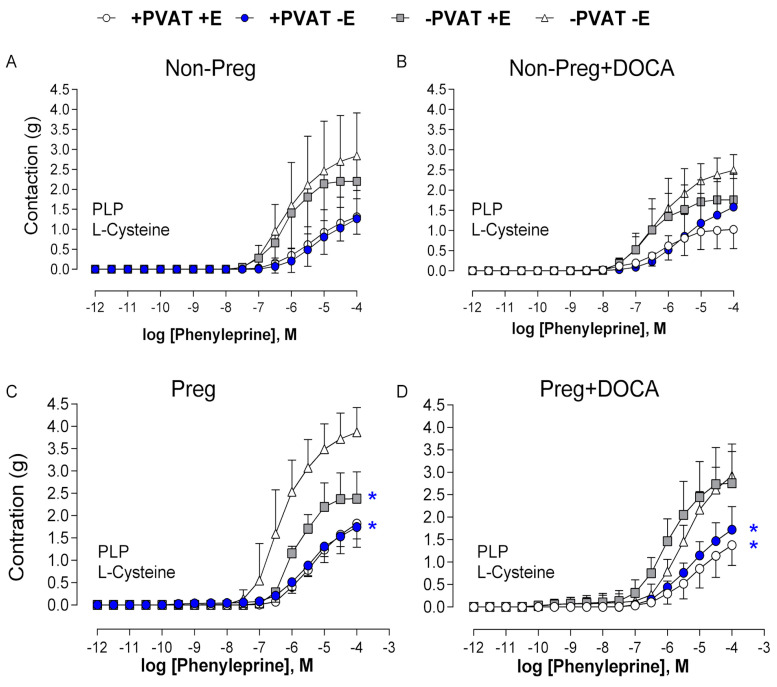
Cumulative concentration responses to phenylephrine after pre-incubation with cofactor (PLP) and substrate (L-cysteine) for H_2_S synthesis in thoracic aortic rings with intact PVAT and endothelium (+PVAT+E), intact PVAT and endothelium removed (+PVAT−E), PVAT removed and intact endothelium (−PVAT+E), and both PVAT and endothelium removed (−PVAT−E) from Non-Preg group (**A**), Non-Preg+DOCA group (**B**), Preg group (**C**), and Preg+DOCA group (**D**). Values are means ± SEM. Symbols indicate significant differences (* *p* < 0.05) *versus* the respective response in aortic rings −PVAT−E (**C**); * *p* < 0.05 *versus* the respective response in aortic rings −PVAT+E and −PVAT−E (**D**).

Pre-incubation with PLP and L-cysteine showed no significant differences in the phenylephrine-induced contractions among all aortic rings with (or without) PVAT and (or) endothelium from the Non-Preg and Non-Preg+DOCA groups (Figure 10A,B; Supplementary Table S3).

Aortic rings from the Preg group with intact PVAT and endothelium, intact PVAT and denuded endothelium, and PVAT removed and intact endothelium showed lower phenylephrine-induced maximum responses compared to the aortic rings in which both PVAT and endothelium were removed (Figure 10C; Supplementary Table S3). However, both aortas in which PVAT was left intact (with endothelium) and denuded endothelium from the Preg+DOCA group showed greater anticontractile responses to phenylephrine compared to the contractions found in the aortic ring preparations in which PVAT was removed, aortas without PVAT and intact endothelium, and aortas without PVAT and denuded endothelium (Figure 10D; Supplementary Table S3).

## 4. Discussion

To induce pregnancy hypertension in the present study, we used the DOCA-salt rat model. The success of the animal model was confirmed by increases in the systolic blood pressure measurements, which were found on the 14th and 19th days of pregnancy in the Preg+DOCA group compared to the respective days and baseline measurements of the other 3 groups. Hence, the present results demonstrate that this rat model resembles human pregnancy hypertension, thereby corroborating previous reports [28,29]. 

The most severe form of the hypertensive disorder of pregnancy is preeclampsia [7]. Preeclampsia has been associated with maternal angiogenic imbalance as well as restriction of placental and fetal development [7]. Thus, we also examined these parameters in the present study. Then, we found reduced placental and fetal weights and decreased litter size in the Preg+DOCA group compared with the Preg group. Thereby, the pregnancy hypertension rat model demonstrates that both fetal and placental developments are impaired, which are also features of preeclampsia. Moreover, in this pre-eclamptic rat model, sFlt-1 levels were increased, and concomitant decreases in PLGF levels were only found in pregnant rats submitted to the DOCA-salt protocol. Thus, our present findings suggest that this rat model induces an angiogenic imbalance of preeclampsia, as previously reported [28,35,38].

*Ex vivo* experiments with aortic rings were performed to assess the individual contributions of PVAT and endothelium to the endogenous H_2_S-induced vasodilation responses stimulated with L-cysteine. In aortic rings with PVAT left intact of the Preg+DOCA rats, vasorelaxation responses induced by L-cysteine were not affected by pregnancy hypertension, whereas impairments were recorded in aortic rings with preserved endothelium and removed PVAT. Furthermore, aortic rings from Non-Preg, Non-Preg+DOCA, and Preg groups showed smaller vasorelaxation responses only in aortic rings in which both PVAT and endothelium were removed, while other aortic rings with PVAT and/or endothelium left intact the vasodilator responses were similar. Importantly, there are few studies examining the role of PVAT on vascular function in normotensive pregnancy [1,39] and in hypertensive pregnancy [27], and, to the best of our knowledge, the present study is the first to investigate endogenous H_2_S-induced vasodilation responses in pregnancy hypertension.

The present results disagree with the work carried out by Al-Jarallah and Oriowo (2016) in rat aorta, in which it was observed that the aortic PVAT lost its anticontractile function during pregnancy [39]. This discrepancy in the results may be related to the anatomic location of the PVAT as well as changes inherent to pregnancy [40]. However, Mann et al. (2015) found that the PVAT of the mesenteric arteries of pregnant rats showed increases in vasodilator responses [1]. In addition, Watts et al. (2020) demonstrated that the thoracic aorta with PVAT enhanced stress relaxation compared with the respective artery without PVAT [41]. These previous studies are in accordance with our present findings. Interestingly, our results in thoracic aorta rings may also suggest that the PVAT of hypertensive and normotensive pregnant rats was responsible for the maintenance of vasodilation. Also, aortic rings with PVAT left intact showed greater vasodilation compared to aortic rings without PVAT that contained an endothelium and/or muscular layers. Thus, the present findings suggest that PVAT-dependent vasodilation may be enhanced by endogenous H_2_S stimulation in pregnancy hypertension, whereas endothelium-derived H_2_S-induced vasodilation showed no additional response compared to the aortic rings in which both PVAT and endothelium were removed.

To confirm the involvement of PVAT-derived H_2_S, we performed pre-incubation with PAG, a CSE inhibitor, followed by PLP incubation, and we then repeated the cumulative concentration curve for L-cysteine. In this experiment setting, the data demonstrated that there was no relaxation in all preparations of Non-Preg, Non-Preg+DOCA, and Preg groups, thus corroborating the findings reported by Fang et al., (2009) [15]. However, interestingly, in the preparations from hypertensive pregnant rats (Preg+DOCA group), there was a slight relaxation in the aortic rings, in which both PVAT and endothelium were maintained, whereas the aortic rings without PVAT and intact endothelium showed no relaxation. Thus, these findings suggest that other H_2_S-producing enzymes may be activated to cause modest vasorelaxant responses induced by L-cysteine, as previously demonstrated [42,43]. By contrast, previous studies with PVAT also showed pro-contractile effects [44], whereas our data reinforce the importance of PVAT-derived H_2_S in maintaining vascular tone in pregnancy hypertension. Hence, our findings suggest that L-cysteine may induce a slight vasorelaxation independently of the H_2_S synthesized by CSE in PVAT and vascular smooth muscle layers of aortas from hypertensive pregnant rats, whereas the endothelium showed no response to L-cysteine in the presence of PAG, which suggests that H_2_S synthesized by CSE is the major enzyme in the endothelium, whereas H_2_S released by PVAT may be synthesized by other enzymes such as CBS [9,43].

As previously demonstrated, metabolic responses to acetylcholine may differ in adipose tissue during pregnancy [45]. Considering that PVAT from non-pregnant or pregnant rats may release substances that distinctively affect acetylcholine metabolism, as previously observed in uterine arteries [46], we then aimed to distinguish the relevance of the roles of PVAT and endothelium in vasodilator responses. To this end, we carried out further experiments to evaluate the acetylcholine-induced responses. We then observed that when the endothelium was preserved in aortic rings without PVAT or with PVAT left intact, there were relaxations with the same magnitude in virgin rats treated (or not) with DOCA-salt (Non-Preg and Non-Preg+DOCA groups). Moreover, the same occurred in the preparations from untreated pregnant rats (Preg group). However, impairments of acetylcholine-induced vasorelaxation responses were found in aortic rings with a preserved endothelium (with or without PVAT) from the Preg+DOCA group, thus confirming that endothelium-derived NO-dependent vasodilation is impaired in pregnancy hypertension [47]. The present results are in accordance with the endothelial dysfunction previously observed in pregnancy hypertension in rats [27]. Thus, our results confirm that NO-dependent vasodilation is impaired and may be associated with pregnancy hypertension-induced endothelial dysfunction [29,47]. Furthermore, our findings demonstrated that aortic rings in which the endothelium was removed but with PVAT left intact do not respond to acetylcholine. Our results may also be corroborated by a previous study that observed a reduction in the vasodilator response to acetylcholine and that was associated with uncoupling of endothelial NO synthase in aortic PVAT of obese mice compared with controls in which PVAT was left intact [48].

To further examine the endothelium-derived NO-dependent vasodilation on endothelial dysfunction-associated pregnancy hypertension, aortic rings were pre-incubated with L-NAME, a non-selective NO synthase inhibitor. Our results demonstrate that no relaxations were found in the aortic rings of all experimental groups, suggesting that NO-dependent vasodilation was blocked by L-NAME, which is in accordance with previous studies [27,49]. Thus, our findings suggest that NO-induced vasodilation is dependent on the endothelium, while PVAT shows no acetylcholine-induced response.

To further understand the involvement of H_2_S and NO in pregnancy hypertension [50], we have analyzed the circulating levels of H_2_S and NO in plasma. We observed that there was a decrease in H_2_S in pregnant rats compared to virgin rats. However, in hypertensive pregnant rats, we noticed an increase in circulating levels of H_2_S compared to normotensive pregnant rats. Moreover, the determination of metabolites of NO in plasma revealed that there was a significant increase in circulating levels of NO in the group of normotensive pregnant rats compared to the other groups, including no increases in the Preg+DOCA group. Hence, the present results suggest that there may be an increased demand for H_2_S in the maternal organism, which results in reduced levels of H_2_S, whereas the production of NO is increased in healthy pregnancy. However, in pregnancy hypertension, we found increases in H_2_S levels and an absence of elevations in NO levels in comparison with the healthy pregnant group, which is in accordance with a previous clinical report [51]. Also, in this previous study, the authors observed that plasma H_2_S increased considerably in preeclampsia compared to the healthy pregnant women group, possibly indicating a compensatory mechanism of the maternal organism in the face of the reduction in NO, which is frequently found in pregnancy hypertension and preeclampsia [52,53,54,55,56].

It is proposed that the decrease in NO bioavailability may be caused, among other factors, by oxidative stress [55,56]. A healthy pregnancy itself is already related to oxidative stress, including several changes in the immune response [57]. The placenta has already been mentioned as one of the main sources of reactive oxygen species (ROS) [57,58]. However, there is previous evidence that further increases in oxidative stress and inflammatory processes occur in gestational hypertension and preeclampsia [59]. Thus, our current results support this concept and demonstrate that increased oxidative stress may be associated with a healthy pregnancy. Also, in the present study, our findings suggest that there may be further elevated levels of plasma malondialdehyde (a biomarker for assessing oxidative stress) in pregnancy hypertension, as previously reported [60,61].

To provide greater robustness to our results, we also measured the stimulated synthesis of H_2_S in the aortic PVAT, in the aorta without PVAT, and in the placenta. We noticed that there was a significant increase in H_2_S production in the aortic PVAT of the Preg+DOCA group compared to the other groups. Placental H_2_S synthesis was also higher in the Preg+DOCA group compared to the Preg group. However, the aorta in which PVAT was removed showed no changes in H_2_S synthesis stimulated with PLP and L-cysteine in all groups, thus corroborating results previously found [27].

Additional experiments were conducted to further confirm the participation of PVAT-derived H_2_S. Then, the vascular tone was challenged to assess the anticontractile effects of H_2_S. Aortic rings were submitted to increasing concentrations of phenylephrine to evaluate the contraction after pre-incubation with PLP and L-cysteine. No significant differences were found in all aortas from the Non-Preg and Non-Preg+DOCA rats, whereas anticontractile effects were observed in aortic rings from the Preg rats in which PVAT and/or endothelium were left intact in comparison with respective aortic rings in which both PVAT and endothelium were removed. However, aortic rings from Preg+DOCA rats showed significant anticontractile responses in aortic rings with both PVAT and endothelium as well as aortic rings with PVAT and denuded endothelium, while loss of anticontractile effect was found in the aortic rings without PVAT and intact endothelium. Then, the present results suggest that in pregnancy hypertension, PVAT-derived H_2_S is important for maintaining anticontractile responses, thus contributing to the regulation of vascular tone in endothelial dysfunction.

The present results demonstrate that vasorelaxation response dependent on endothelium-derived NO is impaired in pregnancy hypertension, indicating that a hypertensive pregnant state renders the endothelium less responsive to NO-induced vasodilation, while PVAT-derived H_2_S-dependent vasodilation is not affected. Thereby, the present results are in accordance with the features of the pregnancy hypertension-associated endothelial dysfunction, as previously demonstrated [47]. Thus, our findings suggest that PVAT-derived H_2_S-dependent vasodilation is preserved in pregnancy hypertension and indicate that hypertensive pregnancy-associated vascular adaptations may have counteracting mechanisms that involve, in part, the vasodilation response dependent on PVAT. Taken together, our findings demonstrate that the PVAT-related anticontractile effect is not affected by pregnancy hypertension and is enhanced by H_2_S stimulation, whereas this response is impaired on the endothelium.

The current findings should be carefully interpreted. Further studies should investigate if the DOCA-salt model of hypertension in pregnant rats could reproduce the morphological changes in the thoracic aorta featured by increases in wall thickness, wall area, and wall-to-lumen ratio, thus suggesting that local changes in the vascular wall occurred in response to increased arterial blood pressure, as previously demonstrated in hypertensive male rats [62,63,64,65,66]. Furthermore, although the present results were demonstrated in the late period of pregnancy in rats, which could resemble the eighth and ninth months of human gestation, further investigation is warranted to examine the role of PVAT-derived H_2_S from a clinical perspective, also considering differences in dietary intake, age, and period of the pregnancy in patients.

## 5. Conclusions

The present study provides evidence that PVAT-derived H_2_S-induced vasodilation may be stimulated with L-cysteine, whereas endothelium-derived H_2_S-induced vasodilation is impaired by the increased oxidative stress related to pregnancy hypertension associated with angiogenic imbalance and fetal-placental growth restriction. Thus, endogenous PVAT-derived H_2_S stimulation with the amino acid L-cysteine may be a clinical alternative to attenuate hypertensive disorders of pregnancy associated with endothelial dysfunction. Further studies are warranted to investigate the potential and beneficial effect of L-cysteine on maternal PVAT in patients with pregnancy hypertension.

## Data Availability

Authors declare that all the data supporting the results of the present study are included in the article.

## Supplementary Materials

The supporting information can be downloaded at https://www.mdpi.com/article/10.3390/antiox12111919/s1, Table S1: Average maternal systolic blood pressure; Table S2: Average maternal, fetal, placental, and biochemical parameters; Table S3: Vascular reactivity measurements.
