# Supplementary material for: Vasodilator Responses of Perivascular Adipose Tissue-Derived Hydrogen Sulfide Stimulated with L-Cysteine in Pregnancy Hypertension-Induced Endothelial Dysfunction in Rats
Source: Antioxidants (Basel). 2023 Oct 26;12(11):1919. doi: 10.3390/antiox12111919 (PMC10669374; doi:10.3390/antiox12111919)
Supplement: Supplementary file 1 [file antioxidants-12-01919-s001.zip › antioxidants-2657962-supplementary.pdf]

Supplementary Materials

Table S1. Average maternal systolic blood pressure (mmHg).

Table .	Non-Preg	Non-Preg+DOCA	Preg	Preg+DOCA
**Baseline**	128 ± 10	126 ± 5	123 ± 5	120 ± 4
**14th**	128 ± 10	124 ± 4	120 ± 7	139 ± 5*
**19th**	128 ± 10	128 ± 5	126 ± 6	161 ± 10*

Values are means ± SEM. Symbols indicate significant differences (*P<0.05) in the Preg+DOCA group *versus* the respective baseline.

Table S2. Average maternal, fetal, placental, and biochemical parameters.

Parameters	Non-Preg	Non-Preg+ DOCA	Preg	Preg+ DOCA
Fetal weight (g)	–	–	3.50 ± 0.07	1.65 ± 0.02*
Litter size (number of pups)	–	–	0.362 ± 0.01	6.9 ± 0.37*
Placental weight (g)	–	–	0.362 ± 0.01	0.239 ± 0.005*
sFlt-1 PLGF ratio	–	–	0.82 ± 0.20	15.56 ± 2.47*
Plasma sFlt-1 (pg/mL)	28.9 ± 2.0	25.2 ± 2.5	79.4 ± 25.4	387.2 ± 35.9*
Plasma PLGF (pg/mL)	–	–	93.3 ± 6.1	29.7 ± 4.8*
Plasma H_2S (μmol/L)	385.2 ± 26.07	455.0 ± 36.1	32.4 ± 3.07*	224.7 ± 38.3#
Plasma NOx (μmol/L)	20.5 ± 1.2	19.20 ± 0.88	29.5 ± 2.22*	18.7 ± 1.52
Plasma malondialdehyde	237.5 ± 17.9	222.6 ± 18.4	341.7 ± 22.8*	439.1 ± 15.7*

Values are means ± SEM. Symbols indicate significant differences (*P< 0.05) versus Non-Preg group; #P < 0.05 versus. Non-Preg and Preg groups.

Table S3. Vascular reactivity measurements.

	+ PVAT + E	+ PVAT -E	- PVAT +E	- PVAT -E
**R_{max} (%)**				
L-Cysteine Non-Preg	82 ± 5*	82 ± 3*	82 ± 3*	67 ± 5
L-Cysteine Non-Preg+DOCA	79 ± 14*	80 ± 14*	79 ± 14*	66 ± 12
L-Cysteine Preg	89 ± 3*	90 ± 16*	86 ± 1*	64 ± 5
L-Cysteine Preg+DOCA	89 ± 3*	90 ± 1*	60 ± 5	64 ± 5
PAG and L-Cysteine Non-Preg	3 ± 3	3 ± 3	3 ± 3	3 ± 3
PAG and L-Cysteine Non-Preg+DOCA	0	0	3 ± 3	3 ± 3
PAG and L-Cysteine Preg	1 ± 0.2	1 ± 0.6	3 ± 3	3 ± 3
PAG and L-Cysteine Preg+DOCA	20 ± 0.3*	18 ± 3*	0	12 ± 0.8*
Acetylcholine Non-Preg	84 ± 3*	7 ± 6	81 ± 5*	3 ± 2
Acetylcholine Non-Preg+DOCA	83 ± 3*	0	87 ± 1*	0
Acetylcholine Preg	92 ± 2*	2 ± 1	92 ± 1*	1 ± 0.3
Acetylcholine Preg+DOCA	77 ± 8*	8 ± 8	69 ± 5*	1 ± 1
PLP + L-Cys and Phenyleprine Non-Preg	1 ± 0.2	1 ± 0.3	2	3 ± 0.5
PLP + L-Cys and Phenyleprine Non-Preg+DOCA	1 ± 0.2	1 ± 0.4	2 ± 0.2	2 ± 0.2
PLP + L-Cys and Phenyleprine Preg	2 ± 0.2	2 ± 0.2	2 ± 0.3	4 ± 0.3
PLP + L-Cysteine and Phenyleprine Preg+DOCA	1 ± 0.1	2 ± 0.2	3 ± 0.4	3 ± 0.2
**pEC_{50}**				
L-Cysteine Non-Preg	7 ± 0.2	7 ± 0.1	7 ± 0.2	7 ± 0.1
L-Cysteine Non-Preg+DOCA	7 ± 0.1	7 ± 0.2	7 ± 0.2	6 ± 0.2
L-Cysteine Preg	7 ± 0.3	7 ± 0.1	7 ± 0.2	5 ± 1
L-Cysteine Preg+DOCA	7 ± 0.2	7 ± 0.1	6 ± 0.2	7 ± 0.2
PAG and L-Cysteine Non-Preg	6 ± 1	6 ± 1	6 ± 1	6 ± 1
PAG and L-Cysteine Non-Preg+DOCA	0	0	6 ± 1	6 ± 1

PAG and L-Cysteine Preg	7 ± 2	6 ± 1	6 ± 1	6 ± 1
PAG and L-Cysteine Preg+DOCA	17 ± 1	14 ± 1	0	20 ± 3
Acetylcholine Non-Preg	7 ± 0.2	5 ± 1	7 ± 0.3	6 ± 0.2
Acetylcholine Non-Preg+DOCA	7 ± 0.1	0	0	7 ± 0.1
Acetylcholine Preg	7 ± 0.3	6	7 ± 0.4	7 ± 0.5
Acetylcholine Preg+DOCA	6 ± 0.6	5	7 ± 0.6	6
PLP + L-Cys and Phenyleprine Non-Preg	5 ± 0.3	5 ± 0.3	6 ± 0.3	6 ± 0.3
PLP + L-Cys and Phenyleprine Non-Preg+DOCA	6 ± 0.3	5 ± 0.2	6 ± 0.2	6 ± 0.3
PLP + L-Cys and Phenyleprine Preg	5 ± 0.1	5 ± 0.1	6 ± 0.1	6 ± 0.1
PLP + L-Cysteine and Phenyleprine Preg+DOCA	5 ± 0.2	5 ± 0.1	6 ± 0.1	5 ± 0.1

Values are means ± SEM. Symbols indicate significant differences (*P<0.05) versus the respective aortic rings - PVAT-E.
